# The Dual Effects of Exosomes on Glioma: A Comprehensive Review

**DOI:** 10.7150/jca.86996

**Published:** 2023-09-04

**Authors:** Maowen Luo, Xingzhao Luan, Gen Jiang, Luxia Yang, Kekun Yan, Shenjie Li, Wei Xiang, Jie Zhou

**Affiliations:** 1 Department of Neurosurgery, the Affiliated Hospital of Southwest Medical University, Luzhou 646000, China.; 2 Department of Neurosurgery, the Affiliated Hospital of PanZhiHua University, PanZhiHua 617000, China.; 3 Southwest Medical University, Luzhou 646000, China.; 4 Sichuan Clinical Research Center for Neurosurgery, Luzhou 646000, China.; 5 Academician (Expert) Workstation of Sichuan Province, Luzhou 646000, China.

**Keywords:** Glioma, exosome, diagnosis, therapy, drug resistance.

## Abstract

Glioma is a frequently occurring type of cancer that affects the central nervous system. Despite the availability of standardized treatment options including surgical resection, concurrent radiotherapy, and adjuvant temozolomide (TMZ) therapy, the prognosis for glioma patients is often unfavorable. Exosomes act as vehicles for intercellular communication, contributing to tissue repair, immune modulation, and the transfer of metabolic cargo to recipient cells. However, the transmission of abnormal substances can also contribute to pathologic states such as cancer, metabolic diseases, and neurodegenerative disorders. The field of exosome research in oncology has seen significant advancements, with exosomes identified as dynamic modulators of tumor cell proliferation, migration, and invasion, as well as angiogenesis and drug resistance. Exosomes have negligible cytotoxicity, low immunogenicity, and small size, rendering them an ideal therapeutic candidate for glioma. This comprehensive review discusses the dual effects of exosomes in glioma, with an emphasis on their role in facilitating drug resistance. Furthermore, the clinical applications and current limitations of exosomes in glioma therapy are also discussed in detail.

## Introduction

Gliomas are tumors arising from glial and neuronal cells in the nervous system, representing 78.2% of all malignant central nervous system tumors in individuals aged over 65 years^1^. The high morbidity and mortality rates are concerning, highlighting the urgent need for effective treatment options. In 2021, the World Health Organization updated the classification and grading of gliomas based on molecular markers, providing significant opportunities for immunotherapy and targeted therapy^2^. However, owing to technical limitations and unclear molecular mechanisms, the standard treatment remains surgical resection, synchronous radiotherapy, and adjuvant TMZ therapy^3^. The generation of TMZ resistance and limited drug delivery through the blood-brain barrier (BBB) significantly impairs the efficacy of treatment, leading to a poor median survival of only 14.6 months in glioma patients^4^. Therefore, the development of novel therapeutic strategies is imperative for mitigating the clinical challenge posed by gliomas.

Exosomes are extracellular vesicles (EVs) with a diameter of 30-150nm and a density of 1.13-1.19g/mL, containing a complex repertoire of metabolites such as lipids, proteins, and nucleic acids^5^. Current research suggests that exosomes are generated through the fusion of multivesicular bodies (MVBs) with the plasma membrane, a process that can be modulated by diverse regulatory mechanisms, resulting in the heterogeneous composition of exosomes^6^, ^7^. MVBs can undergo either lysosomal digestion or exosomal release, depending on their degree of maturation. Exosomes can play both beneficial and detrimental roles in intercellular communication, facilitating the transfer of functional proteins, metabolites, and nucleic acids from donor cells to recipient cells in both physiological and pathological contexts^8^, ^9^. However, accumulating evidence suggests that exosomes are also implicated in the pathogenesis and progression of tumors such as glioma^10^, breast cancer^11^, lung cancer^12^, and colorectal cancer^13^, as well as drug resistance. In the case of glioma, exosomes are crucial in promoting tumor cell proliferation, invasion, migration, angiogenesis, immune invasion, and mediating drug resistance^14^-^16^. Targeting exosomes represents a promising strategy for diagnosing and treating glioma, holding potential to yield innovative breakthroughs.

Exosomes exhibit advantageous characteristics such as broad applicability, ability to readily penetrate the BBB, and low immunogenicity, rendering them an attractive therapeutic strategy for glioma. The rapid advancements in separation and purification technologies, as well as nanotechnology, have presented new possibilities for the clinical diagnosis and treatment of glioma with exosomes. This review provides an overview of the role and mechanism of exosomes in glioma, with a particular emphasis on their contribution to the development of drug resistance. Additionally, the clinical prospects of exosomes in glioma therapy are discussed.

## Exosome biogenesis

Initially believed to function as cellular waste disposal mechanisms, exosomes were first discovered in 1983^17^ and officially named in 1987^18^. Exosomes are a subgroup of extracellular vesicles that can be distinguished from microvesicles (MVs) and apoptotic bodies. Exosomes originate from MVBs which fuse with the plasma membrane^19^ for secretion. The biogenesis of exosomes involves multiple key steps, including substrate sorting, MVB formation, MVB trafficking, the fusion of MVBs with the plasma membrane to generate intraluminal vesicles (ILVs), and the release of exosomes containing the ILVs. This exquisitely orchestrated process is governed by various mechanisms that contribute to the heterogeneous composition and diverse biological functions of exosomes.

According to data from the latest exosome database, exosomes contain diverse types of RNA, proteins, and lipids^20^. Exosomal RNA encompasses mRNA, micro-RNA (miRNA), long non-coding RNA (lncRNA), circular RNA (circRNA), among other species, with crucial implications for the diagnosis and treatment of glioma. Exosomal proteins consist of membrane transport and fusion-related proteins, tetraspanin family proteins, and MVB-associated proteins, characterized by ubiquitination and vitally contributing to the formation of ILVs^21^.

**Figure 1:**
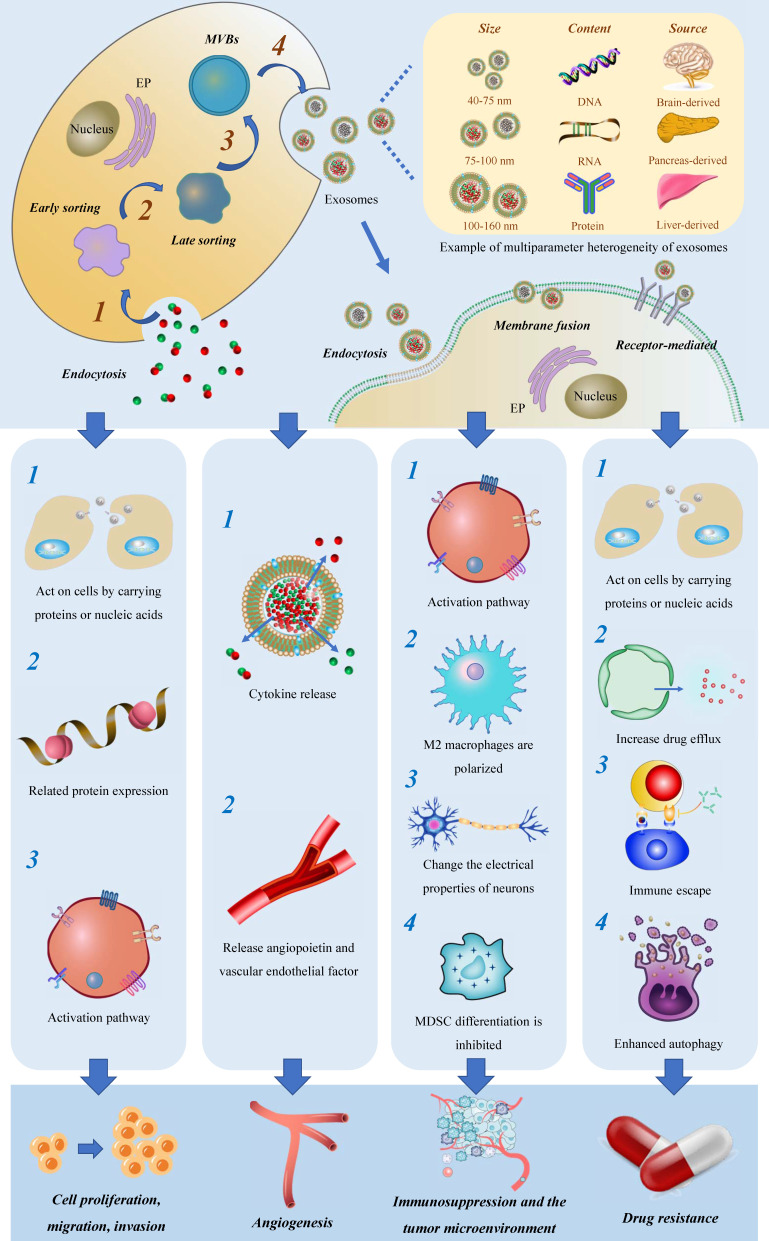
Origin and mechanism of exosomes and their effects on glioma progression.

Current studies on exosomes, as a subtype of extracellular vesicles, have predominantly focused on their function in mediating intercellular communication and transferring materials, thereby participating in a wide array of physiological processes. Exosomes are involved in tissue maintenance and repair^22^, intercellular communication^23^, immune regulation^24^, anti-inflammatory and anti-infective responses^25^-^27^, and facilitating tissue regeneration after injury^28^-^30^. Unlike normal cells, tumor cells secrete exosomes under the control of genetic factors, which maintain cellular homeostasis, evade immune detection, and promote drug resistance^31^ (Figure 1).

Exosomes are derived from diverse human tissues and cells and can be found in various extracellular fluids, including plasma, cerebrospinal fluid, interstitial fluid, lymph fluid, and others^32^. Furthermore, they have the capability to interact with different target cells, facilitating the transmission of biological information^33^. Here we briefly summarize the biological functions and properties of exosomes from different sources (Table 1).

Advances in molecular biology technologies, coupled with advancements in exosome isolation and preparation, have facilitated highly detailed studies of these tiny vesicles. Differential ultracentrifugation (UC) has been widely recognized as a trusted method for isolating exosomes from biological fluids, albeit considered cumbersome in recent times^34^. Sung Jin Back et al. designed CaTiO3:Eu3@Fe3O4 multifunctional nanocomposites for the direct capture and separation of exosomes from complex biological systems, opening up new avenues for portable exosome detection^35^.

Although research on exosomes has made significant progress in recent years, there are still limitations in the separation methods, including their cumbersome nature, low speed, yield, and purity. These limitations hinder the advancement of both basic research and clinical applications of exosomes. As a result, researchers have been actively seeking new, convenient, and efficient separation methods. In 2021, a highly efficient exosome detection method, known as the ultrafast separation system (EXODUS), was introduced. This method utilizes negative pressure oscillations and double-coupled harmonic oscillators to induce membrane vibration, enabling the ultra-efficient purification of exosomes. Excitingly, the EXODUS system allows for automatic labeling-free purification of exosomes from various biological fluids. The development of this method has created vast opportunities for the separation and extraction of exosomes. However, despite its promising potential, the widespread application of this method still requires further popularization in both the research community and clinical settings^36^.

In terms of exosome preparation, biogenic exosomes are typically processed and modified through genetic or chemical engineering^37^. With the development of nanotechnology, attempts to design exosome-mimetic nanoscale structures that replicate the unique features of exosomes are becoming feasible^38^-^40^.

Given the strides made in exosome isolation and preparation technologies, a growing body of research has begun exploring the use of exosomes in tumor treatment. In comparison to artificial nanoplatforms, exosomes offer several advantages, including low cytotoxicity, minimal immunogenicity, high biocompatibility, and readily manipulatable for synthetic modifications. Exosomes have emerged as promising therapeutic agents and gene carriers for potential clinical applications.

## The role and mechanism of exosomes in glioma progression

### Exosomal effects on glioma cell proliferation, invasion, and migration

As carriers of materials, exosomes facilitate the transmission of normal and aberrant substances between cells and the tumor microenvironment (TME), providing an ideal mechanism for the migration and invasion of tumor cells. Furthermore, exosomes act as information carriers, mediating the transmission of abnormal signals through membrane fusion events, leading to altered gene expression and aberrant tumor cell proliferation activity.

The process of cell proliferation involves the modulation of cycle-related proteins, which encompasses various steps such as gene expression, transcription, and translation. In this review, we focus on three main aspects of previous research. Firstly, certain components within exosomes act as essential regulatory factors, directly influencing the proliferation of tumor cells. For example, in sero-derived exosomes, the overexpression of lnc-LINC00470 has been identified as a key regulator of glioma autophagy and proliferation. This molecule binds to miR-580-3p in glioma cells, resulting in the regulation of WEE1 expression and activation of the PI3K/AKT/mTOR pathway, ultimately inhibiting autophagy^14^. There is a strong correlation between this mechanism and the degree of malignant progression and survival time in glioma patients. Secondly, exosomes indirectly contribute to tumor progression through a sorting mechanism. Glioma cells selectively eliminate tumor-suppressive miRNAs via exosomes, which are then transferred to immune cells within the TME. This, in turn, induces a transformation of immune cells into cancer-promoting phenotypes. Conversely, miRNAs that enhance tumor proliferation are selectively retained within tumor cells, continuously promoting cell proliferation. These findings highlight the intricate role of exosomes in tumor proliferation, shedding light on both direct and indirect regulatory mechanisms^41^. What's more, in terms of migration and invasion, several investigations have shown that circNEIL3^42^, miR-3184-3p^43^, miR-3591-3p^44^, miR-148a^45^, miR-15a, and miR-92a^46^ can all be packaged into exosomes via specific pathways and are critical to the migration and invasion of glioma cells. Nonetheless, most of the research remains in the stage of bioinformation analysis and experimental verification, and its mechanism has yet to be effectively proven.

### Exosomes and their impact on glioma angiogenesis

Glioma is often characterized by angiogenesis and hypoxia, which have been linked to unfavorable clinical outcomes^47^. Exosomes have been shown to play a significant role in mediating angiogenesis through various pathways. Firstly, exosomes release cytokines such as IL-8 and VEGF into the TME, which act on endothelial cells to rapidly form peripheral blood vessels. Secondly, exosomes are complex and heterogeneous, especially those derived from tumors, and exhibit significant changes in protein components, often containing angiopoietin and vascular endothelial factors^48^-^50^.

Among these pathways, vasculogenic mimicry (VM) is a unique phenomenon observed in glioma, where independent channels composed of basement membranes without endothelial cells and fibroblasts are formed^51^. The presence of VM can increase the feeding blood flow to hypoxic glioma cells, leading to enhanced glioma cell proliferation^52^. Recent studies by J. Jiang have revealed a downregulation of miR-376b-3p in serum exosomes of malignant glioma patients. Intriguingly, the upregulation of miR-376b-3p in serum-derived exosomes has been shown to enhance the expression of HOXD10, leading to a reduction in glioma cell proliferation and invasion. Additionally, this upregulation impedes the formation of angiogenic mimics. These findings indirectly suggest that serum-derived exosomes have the ability to promote glioma angiogenesis 53. In addition, the delivery of miRNA-29a-3p through exosomes secreted by human MSCs has been found to inhibit VM production and thus impede the progression of glioma^54^.

What's more, hypoxia profoundly affects the repertoire and composition of proteins present in exosomes derived from glioma cells. Notably, selective upregulation of protein-lysine 6-oxidase (LOX-6), VEGF, thrombospondin-1 (TSP1) and other proteins has been observed, which converge in the tumor neovascularization region and may serve as key players in tumor angiogenesis^55^.

### Exosomes induce the formation of glioma immunosuppressive microenvironment

Immune cells, particularly macrophages and monocytes, comprise the majority of immune cells within the TME^56^. Increasingly, studies have revealed that exosomes are key mediators of the critical link between glioma cells and the surrounding microenvironment^57^. Simultaneously, exosomes are closely associated with immune tolerance in glioma. They not only aid tumor cells in evading immune surveillance and creating an environment conducive to tumor cell generation through direct inhibition of immune cells, but also play adverse roles in immune regulation within the body. They achieve this by transmitting inhibitory signals that promote the establishment of immune tolerance in glioma cells.

Exosomes play a pivotal role in inducing the formation of an immunosuppressive microenvironment through three major mechanisms. Firstly, they stimulate the polarization of M2 macrophages, contributing to the maintenance of chronic inflammation and increased tumor aggressiveness. This polarization can be achieved through the delivery of tumor suppressor miR-3591-3p^44^ and miR-1246^58^, ^59^ by tumor-derived exosomes. Secondly, exosomes facilitate the generation of myeloid suppressor cells (MDSC), which play a critical role in establishing immunosuppressive microenvironments and assisting tumors in evading host immune responses^60^. MiR-29a and miR-92a promote the proliferation of MDSC by targeting high-mobility group box transcription factor 1 (Hbp1) and protein kinase cAMP-dependent type I regulatory subunit alpha (Prkar1a), respectively^61^. Furthermore, the presence of stem-like brain tumor initiation cells (BTIC) with enhanced resistance to radiation and chemotherapy contributes to the poor prognosis of glioblastoma. Exosomes released by BTIC carry tenascin-C (TNC) and have been shown to inhibit T cell activity^62^.

In several clinical studies, mounting evidence suggests that the TME has a crucial role in epileptic seizure onset in glioma patients. Recent findings suggest that exosomes can be released by tumor cells through synapses and carry out abnormal information transfer between neurons. As a result, specific rearrangements occur in neuronal connections, exerting a far-reaching influence on neuronal network activity and synchronization, leading to cognitive decline in glioma patients, and severe cognitive impairment in more severe cases^63^. These studies provide valuable insights into the debilitating complications yielded by glioma, and hold considerable significance in terms of enhancing the quality of life of affected patients.

### The association between exosomes and drug resistance in glioma

Surgical treatment combined with radiotherapy and TMZ is one of the most frequently employed methods for glioma treatment. TMZ is an oral alkylating agent that directly damages tumor cell DNA by attacking the N-7 and O6 sites of guanine, as well as, the N-3 sites of adenine. The formation of O6-methylguanine (O6-meG) is a crucial product that plays a role in inducing apoptosis^64^. Various factors, such as DNA damage repair, immune stress response, abnormal expression of proto-oncogenes and tumor suppressor genes, contribute to TMZ resistance. At present, O6-methylguanine-DNA methyltransferase (MGMT) is the most common marker of TMZ resistance, with its presence leading to resistance to TMZ by removing TMZ-induced alkylation^65^. Exosomes have a dual influence on glioma drug resistance. On one hand, they can promote drug resistance, even inducing the transition of TMZ-sensitive cells into drug-resistant ones. On the other hand, certain exosomes possess the ability to reverse TMZ-resistant glioma cells, rendering them sensitive to treatment. In this discussion, we will explore the former scenario first.

**Figure 2:**
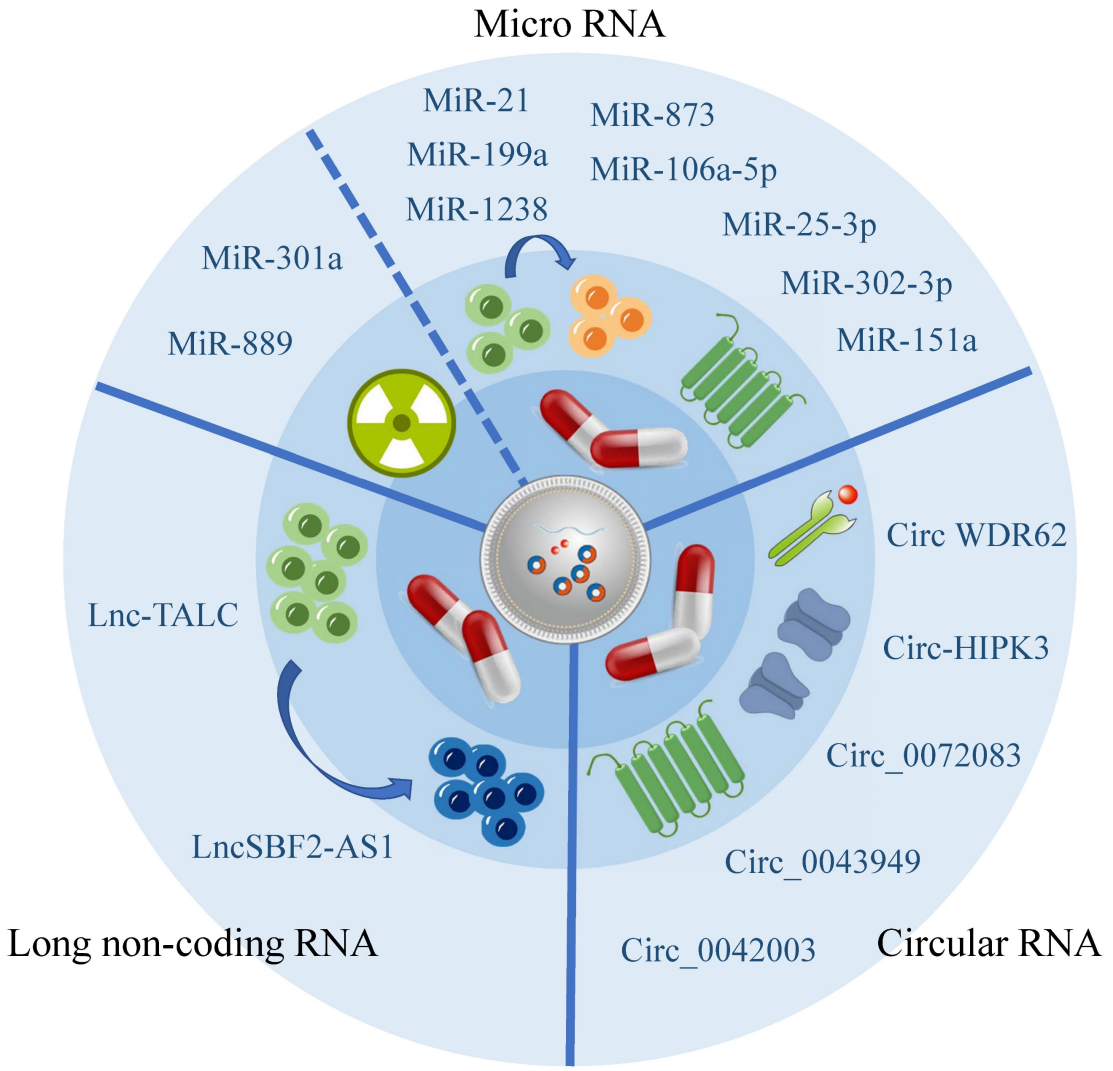
Exosomes are associated with glioma drug resistance.

Exosomes can carry a diverse range of substances, with the direct or indirect targeting mediated by RNA being one significant contributor to drug resistance, according to current research^66^. Numerous studies have highlighted the importance of exosome-associated ncRNA in regulating the chemo- and radiation resistance of glioma cells through various pathways, demonstrating their strong connection to the development of glioma resistance (Figure 2, Table 2).

#### Exosomal miRNA influence glioma drug resistance

MiRNA is a class of regulatory molecules that are around 22 nucleotides long, and processed by RNA precursors, playing multiple functions such as down-regulating gene expression^67^, and transmitting information^68^.

Recent studies have been summarized, revealing that miRNA has been shown to decrease the vulnerability of glioma cells towards TMZ. Additionally, certain miRNAs have the ability to penetrate exosomes released by TMZ-resistant cells and be absorbed by TMZ-sensitive cells, resulting in the transmission of TMZ resistance. Exosomes originating from hypoxic glioma cells have demonstrated the capability to diminish PTEN expression through the transportation of miR-106a-5p, consequently reducing the sensitivity of glioma cells to TMZ^69^. Furthermore, bioactive miR-1238 and miR-25-3p have been observed to be incorporated into exosomes released by TMZ-resistant cells, thus having the potential to be absorbed by TMZ-sensitive cells and propagate TMZ resistance^70^, ^71^.

In addition to the increased chemotherapeutic resistance associated with TMZ, Xiao Yue's research has also identified that exosome miRNA is associated with a decrease in the effectiveness of radiotherapy. Specifically, exosome miR-301a has been found to activate the Wnt/ beta-catenin signaling pathway by targeting TCEAL7 under hypoxic conditions, which reduces radiation sensitivity and ultimately minimizes the radiotherapy effects of glioma^72^.

#### Exosomal lncRNA and its influence on glioma drug resistance

LncRNA are a class of ncRNA that are over 200 nucleotides in length. They are characterized by low stability, poor conservation, and are closely associated with the occurrence and progression of several cancers^73^. Some studies have demonstrated that exosomes can shield lncRNA from degradation^74^. In glioma, exosome lncSBF2-AS1 is secreted from glioma cells into the TME to promote the development of drug resistance in tumors^75^. This mode of material transfer significantly complicates the treatment of glioma.

#### Exosomal circRNA Influence Glioma Drug Resistance

CircRNA hold a unique annular structure, which distinguishes them from miRNA and lncRNA. With the rapid advancements in gene sequencing and high-throughput bioinformatics, their crucial role in diverse physiological and pathological processes, including cell proliferation, growth, differentiation, and senescence, has been established^76^.

CircRNA has been increasingly recognized as having a significant impact on glioma drug resistance. However, its effect on drug resistance is primarily indirect, as it affects miRNA expression. This suggests that exosomal miRNA plays a crucial role in the development of glioma drug resistance. The Warburg effect plays a significant role in promoting the release of exosomal circ_0072083 from TMZ-resistant glioma cells, which elevates glioma resistance to TMZ by selectively targeting miR-5-1252p^77^. Likewise, exosomal circWDR62 from TMZ-resistant glioma cells participates in transmitting resistance between TMZ-sensitive and non-sensitive cells through the miR-370-3p/MGMT axis^78^. Additionally, exosomal circ-HIPK3 from TMZ-resistant glioma cells regulates glioma via the miR-421/ZIC5 axis, thereby enhancing its drug resistance^79^.

Besides its indirect impact on glioma drug resistance through miRNA, circRNA may also have an influence on other aspects. However, further research is required to explore these potential roles. Recent research has identified heparinase as a key regulator of exosomal secretion that plays a crucial role in the development of drug resistance. This mechanism may be attributed to the delivery of hsa_circ_0042003 through exosomes^80^.

#### Other Implications of Exosomes in Glioma Resistance

Apart from the well-established role of exosomal RNA in drug resistance, various other factors continually influence the development of glioma resistance. In recent years, it has been observed that exosomes are capable of transferring therapeutic drugs from within tumor cells to the extracellular environment, thereby rendering them ineffective. Moreover, exosomes facilitate the formation of an effective fibrous barrier by inducing fibroblast response, which prevents therapeutic drugs from reaching specific sites.

As mentioned earlier, MGMT is an essential biomarker in glioma drug resistance prediction. Barbara Oldrini et al. have demonstrated the significance of MGMT rearrangement in inducing drug resistance, which effectively addresses the limitations of grouping MGMT methylation. Furthermore, they have successfully detected the fusion gene after MGMT rearrangement in tumor-derived exosomes, thereby offering a promising avenue for future medication guidance through exosome detection^81^. Rajshekhar A Kore et al. have reported a smaller volume of tumor-derived exosomes in hypoxic environments, which may be attributed to exosomal metabolism. This finding may help elucidate the intricate relationship between drug resistance and hypoxia, but further research is required to uncover the underlying mechanism^55^.

In this review, we have primarily focused on the impact of non-coding RNA in exosomes on glioma drug resistance. However, we are also intrigued by the potential involvement of proteins and lipids in exosomes. Despite our extensive literature review, we have yet to establish a definitive link between these components and the development of glioma resistance. This area warrants further investigation in future studies due to the complex and diverse nature of proteins and other metabolites in exosomes.

## Clinical application of exosomes in glioma

### Exosomes for diagnosis and prognosis of glioma

Brain tumors present distinct diagnostic challenges due to the BBB. Hence, conventional circulating biomarkers, including circulating tumor cells, are often not of diagnostic value in glioma. Exosomes, a type of extracellular vesicle, carry rich genetic information and are widely found in various bodily fluids, presenting as potential markers for tumor diagnosis and prognosis^82^, and as an auxiliary modality for existing imaging techniques to improve the early diagnosis of glioma^83^.

From a diagnostic perspective, exosomes play a significant role in determining the pathological grade of glioma. Notably, exosomes derived from serum samples of glioma patients exhibit abnormal overexpression of miR-210, and this expression level increases with the progression of glioma grade^84^. Additionally, the elevation of miR-301a levels is associated with higher pathological grades and lower Karnofsky physical status (KPS) scores. In serum samples, miR-301a is predominantly found in exosomes^85^. Furthermore, glioma cells release exosomes enriched with the cancer-associated lncRNA, lncSBF2-AS1, which reshapes the TME and contributes to the development of chemotherapy resistance. Therefore, the presence of lncSBF2-AS1 in serum may serve as an indicator of refractory glioma^75^.

In terms of prognosis, tumor-associated macrophages (TAMs) are the predominant cell population within the glioma microenvironment and play a crucial role in glioma initiation and malignant progression^86^, ^87^. Recent studies have demonstrated that circNEIL3, facilitated by hnRNPA1B2, can be packaged into exosomes and delivered to infiltrating TAMs. This process leads to the acquisition of immunosuppressive properties by TAMs, as circNEIL3 stabilizes IGF3BP3, thereby promoting glioma progression. The detection of exosomal circNEIL3 levels may serve as a prognostic indicator for glioma^42^. Additionally, exosome-derived miR-1246 has been shown to impact the polarization of M2 macrophages. Notably, this microRNA is enriched in the cerebrospinal fluid of glioma patients and its levels decrease after tumor resection, indicating that it may also be one of the prognostic indicators of glioma^58^.

It is noteworthy to consider the relevance of exosome detection in predicting the efficacy of radiotherapy. Zihuang Li conducted a study examining 34 genes that exhibited differential expression, with 11 of them being associated with glioma. Notably, after radiotherapy, a significant reduction in miR-574-3p was observed. This finding suggests that miR-574-3p may serve as a promising biomarker candidate for monitoring the effectiveness of radiotherapy^82^.

Exosomes serve as high-quality, nanoscale information transmitters that are ubiquitous in cerebrospinal fluid and blood^88^. While we acknowledge the significant potential of exosomes as biomarkers, it is important to highlight that current studies only demonstrate that the elevation of exosome levels is a consequence of tumorigenesis, rather than a causative factor. The evidence regarding exosomes as a cause of tumorigenesis necessitates further examination and discussion.

### Exosomes as targeted therapy vectors for glioma

Glioma, a central nervous system tumor, presents a poor prognosis, primarily due to several factors. Firstly, the presence of the BBB limits the efficacy of drugs due to their inability to directly reach the tumor area. Secondly, glioma exhibits resistance to lipophilic drugs such as TMZ, further hindering appropriate treatment^89^. Lastly, the therapeutic effect of a single drug may be insufficient, leading to incomplete treatment and disease recurrence. Consequently, the exploration of more effective and accurate treatment methodologies that improve glioma's therapeutic effect and prognosis is critical.

Nano-drugs have shown promise in improving chemotherapy drugs' targeted accumulation, biological distribution, and penetration efficiency, providing significant clinical prospects for diagnosing and treating next-generation tumors^90^. Exosomes, nanoscale structures with bilayer membranes, carry low immunogenicity, high protective ability, and strong penetration capacities across the BBB^91^. Current research has reported the use of exosomes as tumor drug hosts, demonstrating clinically relevant achievements. Exosomes loaded with therapeutic molecules serve as promising biomolecules that can enhance targeted accumulation, improve drug efficacy, and reduce side effects. Hence, exosomes may hold great potential in revolutionizing current tumor therapy approaches (table 3).

Despite exosomes' potential as loading materials, they present several limitations, such as time-consuming purification procedures, low yield, and difficulties related to large-scale production. Recent comparative studies have demonstrated that biomimetic nanovesicles (BNVs) exhibit similar targeting properties, BBB penetration, and load capacity as exosomes^92^. To some extent, BNVs may serve as substitutes for exosomes in industrial production and large-scale applications, creating new opportunities for future exosome research.

### Exosomes in reversing therapeutic resistance in glioma

TMZ is an FDA-approved first-line chemotherapy drug for glioma and a DNA alkylating agent^3^. However, some glioma patients experience drug resistance after TMZ chemotherapy, hindering treatment efficacy. DNA repair, transporter expression, and MGMT overexpression commonly contribute to drug resistance development^93^. As previously mentioned, exosomes play a dual role in glioma drug resistance. In this section, we will specifically explore the capacity of exosomes to convert drug-resistant glioma cells into drug-sensitive ones.

To overcome chemotherapy resistance in glioma, exosomes can be utilized as drug carriers. Examples of this include the design of a double-receptor-specific exosome loaded with TMZ and BG, which aims to repair O-alkylation damage caused by TMZ^94^. Another approach involves the direct delivery of miR-199a to glioma cells through exosomes, which are derived from MSCs. This delivery inhibits glioma cell proliferation, invasion, and migration while enhancing chemotherapy sensitivity^95^. Similarly, the loading of exogenous miR-151a into exosomes emerges as a potential strategy to reverse glioma resistance^96^. Recognizing the clinical significance of exosomes, Fawad Ur Rehman investigated the utilization of exosomes (BMSCEXO) derived from allogeneic bone marrow MSCs for TMZ-resistant glioblastoma. These exosomes were employed as carriers for heme oxygenase-1 (HMOX1) specific short peptides (HSSP) and siRNA^97^.

Collectively, these exosome-related molecular biological findings hold promise as adjuvant therapy with TMZ chemotherapy to enhance the therapeutic efficacy of refractory gliomas.

### Other clinical applications of exosomes in glioma

Nucleic acids and proteins are fundamental regulatory molecules that affect almost every aspect of the tumor process. Exosomes serve as carriers for these major molecules, providing a viable approach for their transportation^98^. Current studies aim to block abnormal exosome transmission to treat tumors, including glioma, by inhibiting exosome production, release, and reuptake^41^, ^58^, ^99^.

Tumor-derived exosomes carry antigens that induce immune responses^100^. Ning Bu et al. discovered that DC-derived exosomes produce cytotoxic effects against autogenous tumors, while exosomes obtained from ascites and pleural effusion of ovarian cancer patients demonstrate anti-tumor therapy effectiveness^101^. These findings suggest that exosomes also relate to current research on tumor immunotherapy, which is an area in need of further exploration.

## Future perspectives

**Figure 3:**
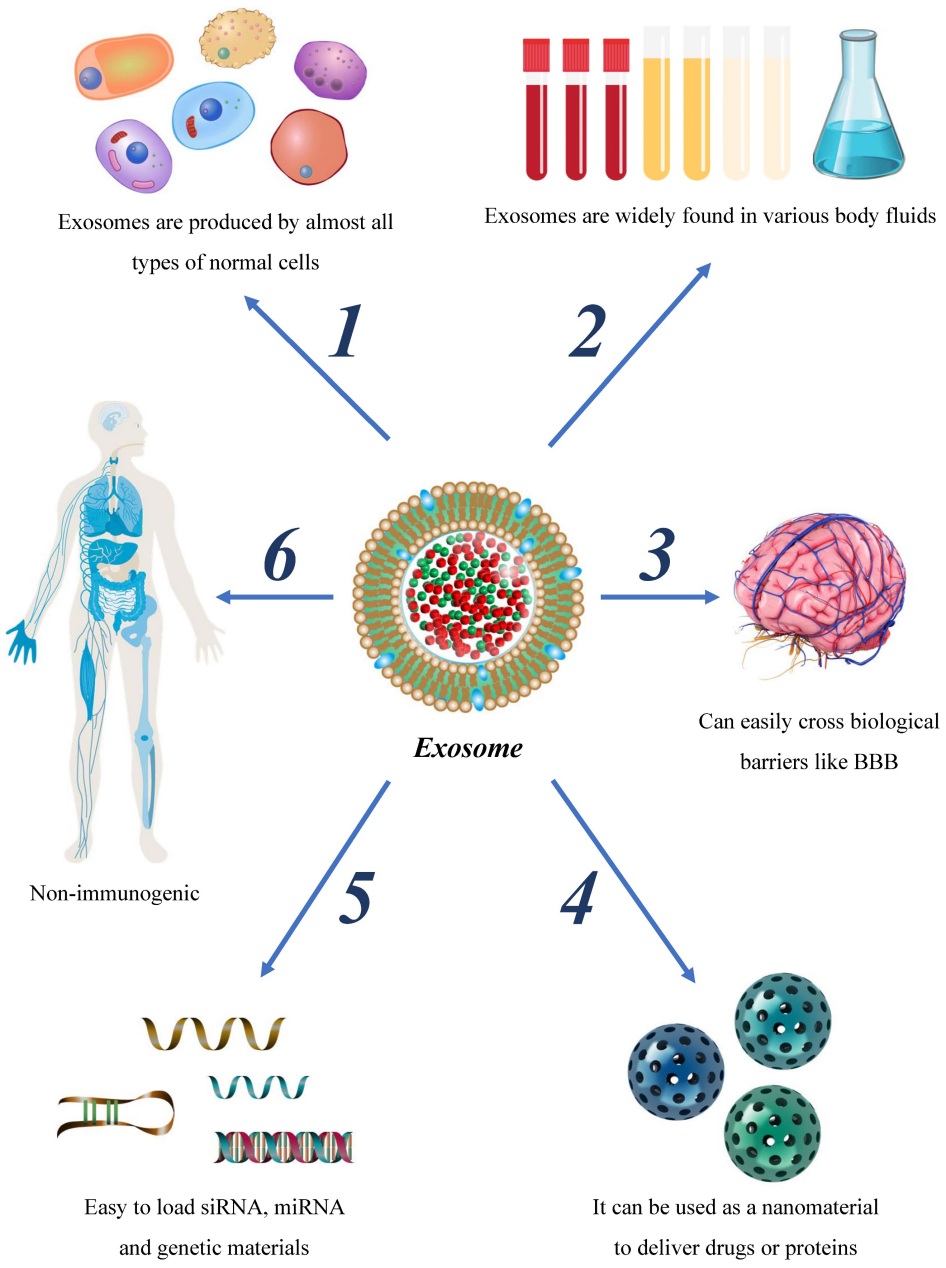
Advantages of exosome as the therapeutic nano-carrier.

Glioma is a prevalent central nervous system neoplasm with a high mortality rate. Although combined therapies, including surgical excision, radiotherapy, and chemotherapy have been employed in recent years, refinements are required to optimize their therapeutic efficacy. Exosomes, a type of extracellular vesicle characterized by their bilayer membrane structure, have unique properties that make them important in the regulation of glioma. While the impacts of exosomes on glioma are multifaceted, exhibiting effects on glioma migration, invasion, immune regulation, drug resistance, and other aspects, many of their underlying mechanisms remain elusive. Considering the molecular size, molecular origin, and immune properties of exosomes, these vesicles possess unique advantages over other approaches for the diagnosis and treatment of glioma (Figure 3). In this study, we comprehensively review the origin and mechanisms of exosomes, their influence on glioma progression, and their role in the treatment of glioma.

The utilization of exosomes for clinical diagnosis and therapy remains a challenging task based on current research. The foremost challenge is the difficulty in achieving high levels of exosome purity through currently available methods and techniques. Furthermore, as exosomes are natural extracellular vesicles, their production is currently limited, and strategies to significantly increase their production require further investigation. Additionally, the transport mechanism of miRNA within exosomes has yet to be fully elucidated, and the cytotoxicity and side effects of miRNA-loaded exosomes have yet to be thoroughly studied. Ultimately, the clinical application of exosomes lacks standardization, and inappropriate usage may result in irreversible adverse effects on patient outcomes. These issues significantly impede the clinical application of exosomes and hamper their potential efficacy in the treatment of glioma.

Continuous research and exploration on the loading mechanism, cytotoxicity, and standardized use of exosomes remain necessary for future developments. Innovative approaches, such as simulating the natural structure and biological behavior of exosomes and developing bio-cell derived nanocarriers yielding higher quantities, could potentially overcome current limitations of low exosome production and accessibility. Further investigating the effects of exosomes on glioma angiogenesis, TME, and immunosuppression is necessary to meet the varying needs of patients and achieve targeted treatment.

Overall, exosomes provide a promising avenue for targeted and personalized therapy in the diagnosis and treatment of glioma. In conclusion, while exosomes have both benefits and drawbacks in relation to gliomas, we must strive to leverage their advantages to advance glioma treatment and mitigate their adverse influence on malignant progression.

## Figures and Tables

**Table 1:** The biological functions and properties of exosomes from different sources.

Exosome source	Present site	Biological function	Clinical significance
Normal cells^102^	Every part	Intercellular communication	/
Tumor cells^13^, ^103^	Cerebrospinal fluid, serum	It plays an important role in tumor growth, metastasis, and formation of tumor immunosuppressive microenvironment	It can be used as the target of tumor therapy and drug carrier
Mesenchymal stem cell (MSC)^104^, ^105^	Bone marrow, umbilical cord blood, placental tissue, adipose tissue	Substance transport, immune regulation, maintenance of homeostasis, tissue repair	It can be used as the main source of exosomes
Macrophage^106^	Bone marrow and serum	Immunoregulation	It plays an important role in tumor invasion and drug resistance formation
Dendritic cell (DC)^107^, ^108^	T cell dependent region of peripheral immune organs	Immunoregulation	It plays an important role in tumor invasion and drug resistance formation
Virus infected cell^109^, ^110^	From the site of the virus gradually spread to all parts	Transfer of bioactive ingredients associated with viral infection	An important therapeutic target for controlling certain virus-infected diseases

**Table 2:** Occurrence and development of exosomal RNA and glioma resistance.

Source	Exosomal-RNA	Mode of action	Medicine	Drug susceptibility	Clinical significance
Hypoxic glioma cells	MiR-106a-5p^69^	By regulating PTEN/Akt signaling	TMZ	Reduce	Provide new ideas for targeted therapy
MSC	MiR-199a^95^	Down-regulating AGAP2 inhibits glioma progression	TMZ	Improve	Provide new molecular targets
TMZ resistant glioma cells	MiR-1238^71^	From non-sensitive cells to sensitive cells	TMZ	Reduce	Promising molecular targets
TMZ resistant glioma cells	MiR-25-3p^70^	Target the regulation of FBXW7	TMZ	Reduce	Prognostic marker
TMZ resistant glioma cells	MiR-151a^96^	Exogenous miRNA is transferred through exosomes	TMZ	Improve	Used for TMZ combined therapy
TMZ resistant glioma cells	LncSBF2-AS1^75^	Remodel the TME	TMZ	Reduce	As a diagnostic marker of refractory glioma
TMZ resistant glioma cells	Circ_0043949^111^	Up-regulated ITGA1 axis of oncogene	TMZ	Reduce	Providing potential molecular targets
TMZ resistant glioma cells	Circ_0072083^77^	Regulate the expression of NANOG and ALKBH5	TMZ	Reduce	Important targeting marker
TMZ resistant glioma cells	CircWDR62^78^	Adjust the miR-370-3p/MGMT axis	TMZ	Reduce	Targeting markers and prognostic markers
Glioma cells	Circ_0042003^80^	Mediated by heparinase	TMZ	Reduce	To provide ideas for the development of new treatment strategies
TMZ resistant glioma cells	Circ-HIPK3^79^	Regulate the miR-421 / ZIC5 axis	TMZ	Reduce	Improve the therapeutic effect of TMZ

**Table 3:** Application of exosomes in drug or gene loading.

Load material	Carrier	Clinical significance
Superparamagnetic iron oxide nanoparticles (SPIONs) and curcumin (Cur)	RGE-Exo-SPION / Cur^112^	As a targeting ligand of NRP-1, it is used for targeted imaging and therapy of glioma cells
DOX	Bioinspired neutrophil-exosomes (NEs-Exos) system^113^	It has strong neutrophil chemotaxis and BBB penetration
TMZ and O6-benzylguanine (BG)	Dual-receptor-specific exosomes^94^	The activity of O6-alkylguanine-DNA alkyl transferase (AGT) was inhibited by transferring alkyl to Cys145, which played a role in enhancing TMZ
Panobinostat and PPM1D-siRNA	A biomimetic nano drug delivery system (cEM@DEP‐siRNA)^114^	For targeted therapy of diffuse intrinsic pontine glioma (DIPG) caused by PPM1D mutation
Selumetinib	Exosomes derived from U87MG cells^115^	Targeting gliomas without cytotoxicity
Magnetic nanoparticles (MNPs)	engineered exosomes^40^	It provides a new idea for enhancing iron death in collaborative GBM therapy.
DOX and PTX	Exosomes were loaded through a microfluidic device (Exo-Load)^116^	Further optimized potential drug loading devices for exosomes were designed
DOX	Endothelium-derived exosomes^117^	Immunogenic chemotherapy for glioma
TMZ and Dihydrotanshinone (DHT)	Reassembly-exosomes (R-EXO)^118^	It is used to reverse TMZ resistance

## Abbreviations

MiRNA: micro-RNA
LncRNA: long non-coding RNA
CircRNA: circular RNA
TMZ: temozolomide
BBB: blood-brain barrier
EV: extracellular vesicle
MVB: multivesicular body
MV: micro vesicle
ILV: intraluminal vesicles
LOX-6: protein-lysine 6-oxidase
VEGF: vascular derived endothelial factor
TSP1: thrombospondin-1
GAM: glioblastoma-associated microglia
hBMEC: human brain micro vessel endothelial cell
MDSC: Myeloid-derived suppressor cell
BTIC: Brain tumor initiating cell
TNC: tenascin-C
MGMT: O6-methylguanine-DNA methyltransferase
ncRNA: non-coding RNA
DOX: doxorubicin
Nes-Exo: Bioinspired neutrophil-exosome
DHT: Dihydrotanshinone
Cur: curcumin
SPION: Superparamagnetic iron oxide nanoparticle
AGT: O6-alkylguanine-DNA alkyl transferase
BG: O6-benzylguanine
DIPG: diffuse intrinsic pontine glioma
MNP: magnetic nanoparticle
PTX: paclitaxel
BNV: biomimetic nanovesicle
HMOX1: Heme Oxygenase-1
MSC: mesenchymal stem cell
DC: dendritic cell
siRNA: small interfering RNA
TME: tumor microenvironment
